# Boosting awareness on healthy habits among school children in north Gujarat, India

**DOI:** 10.6026/97320630018786

**Published:** 2022-09-30

**Authors:** N Sivasubramanian, B Mahalakshmi, Sandeep Garg, Patel Shahin Aiyubdaud, Bepari Soma, KJ Shaijo, Robin Abraham, Bhasara Kalpana Ramji

**Affiliations:** 1 Department of Psychiatric Nursing, Nootan College of Nursing, Sankalchand Patel University, Visnagar, Gujarat - 384315, India; 2 Department Paediatric Nursing, Nootan College of Nursing, Sankalchand Patel University, Visnagar, Gujarat - 384315, India; 3 Tantia University, Sri Ganganagar, Rajasthan, India; 4 Sankalchand Patel University, Visnagar, Gujarat - 384315, India

**Keywords:** Awareness, healthy habits, school children, snake, ladder game

## Abstract

Hygiene is the science of health and its maintenance. Hygiene status of children is an index of national investment in the development of its man power. It is influenced by social, familial and individual factors as well as the children's knowledge of health on personal hygiene, comfort and basic needs. The utility of games is as a teaching strategy of health professionals. The main objectives of the study were to assess existing level of awareness regarding healthy habits among school children and to check the effect of Modified snake & ladder game in improving the awareness of children regarding healthy habits. Research design selected for this study was pre experimental one group pretest posttest design with a sample size of 60. Samples of the study were given opportunity to play and earn awareness through the modified snake & ladder game. Their awareness was assessed before and after the game section. Data was analyzed by using descriptive and inferential statistics such as mean, standard deviation and chi square test. In data analysis the mean of pre-test score was 13.83 and mean of post-test score was 18.63. The mean difference was 4.8. The standard deviation of pre-test stress score was 01.07 and post-test stress score was 01.60. The calculated’ 't' value was 21.24, which was greater than the table value(1.67).The study concluded that snake and ladder game was effective in improving the awareness of school children on healthy habits.

## Background:

One of the most important times for a person's health and development is their childhood. The majority of children in India experience an impoverished upbringing beginning at birth. Children who attend school make up one-fifth of the population and are the country's future. Therefore, it is essential to monitor the health of school children [1]. Childhood lays the groundwork for lifelong responsibility for maintaining personal hygiene, which is crucial for a healthy childhood, healthy adulthood, and the formation of positive health values. During this time, one can build healthy habits [2]. Young children who practice poor personal hygiene and inadequate sanitation habits are more likely to contract infectious diseases. The evaluation of schoolchildren's hygiene literacy, habits, and effectiveness of school-based hygiene initiatives still needs to be done holistically [3]. It is more likely that habits acquired during this time will last into adulthood. Due to the fact that children spend the majority of their learning time in schools, it has long been recognized that schools are the best place to administer health-related initiatives. A review of numerous studies reveals that after intervention, schoolchildren's knowledge, attitudes, and personal hygiene behaviours improved [4], [5]. Schoolchildren's poor health is a result of their lack of knowledge about the advantages of maintaining good personal hygiene. Future generations can be stronger and healthier by putting an emphasis on children today and providing them with the tools and information to modify behaviour. A helpful initiative in this direction might be a school-based health education programme [2]. To increase hygienic and sanitary practices, many initiatives should incorporate concerned and coordinated health education activities [1]. In comparison to teaching according to everyday activities using traditional techniques, school health programmes based on hygiene and sanitation education have several benefits. Additionally, it provides youngsters with the chance to express their creativity and learn from one another about routine tasks that are focused on maintaining sanitary standards (WHO, 2010). It is possible to teach children their personal hygiene habits in a playful manner. Children can be motivated by using charts, graphs, comedy, stickers, puppets, games, or music, among other things. Games can be a creative and difficult instructional tool. They have been utilized as a teaching approach for years, encouraging involvement and self-learning in both child and adult education. Games seem to improve retention and application by involving repetition and allowing key themes to be repeated [6]. It was decided to identify and evaluate the effects of water, sanitation, and hygiene interventions in schools by a systematic assessment of the literature. Thirteen studies show that knowledge, attitudes, and actions have changed, including the use of soap when washing one's hands. This review study demonstrates that educational interventions among schoolchildren can have a significant impact on their knowledge and behaviour with regard to healthy behaviours [7]. Children's visual alertness was raised through a game-based intervention programme that included visual coding, which made it easier for them to comprehend oral health recommendations. Children had a good memory for the instructions and maintained them for a longer time. This was demonstrated by a notable rise in knowledge scores (28.1%) and a fall in debris scores (measured right after the intervention programme) [6]. A modified version of the board game Snakes & Ladders was given to African primary school students. After playing the game, compared to before playing the game, the children were evaluated to see if they were able to properly answer more questions about Taeniasaginata and Taeniasolium transmission and control. A pre- and post-test assessment was given to the kids before and after they played the game just once. Before playing the game, there were 40.3% of the total accurate answers; after playing the game, there were 58.8% of the total correct answers. The use of this well-known board game as a teaching tool for health-related lessons with school-aged children has shown to be promising [8]. Therefore, it is of interest to evaluate the efficacy of modified snake & ladder game in improving awareness of school children regarding health habits to be followed.

## Methodology:

The effectiveness of a modified version of the snake and ladder game in raising schoolchildren's knowledge of good health practices was assessed using a quantitative research methodology. With a sample size of 60, the pre-experimental one-group pretest-post test design was chosen for this investigation. The data, which includes demographic information, was gathered through the use of questionnaires. A closed-ended questionnaire was used to gauge children’s awareness and adherence to healthy behaviors. The scoring of questionnaire were divided in to three, (1-8) = Average, (9-16) = Good, (17-25) = very good. The classic snake and ladder game was altered with the addition of personal hygiene principles, such as dental hygiene, bathing, hair cleaning, nail trimming, washing hands, washing clothes, wearing foot wear, sleeping hygiene, food and water hygiene, and ear hygiene. The researcher provided a personal hygiene rationale for each box. Three questions are posed when a youngster bumps against the ladder, and he or she climbs the ladder when the questions are correctly answered. The researcher also provided an explanation for the boxes that were left out. If the answers were incorrect, players were forced to play through the boxes again and were not able to climb the ladder. Three questions are posed in response to a child striking the snake. They were instructed to resume the game from the same box if they provided the right response. If they provided incorrect information, they were bitten by the snake, forced to descend, and forced to start the game over from the snake's tail. This intervention was applied to the children for 10 days by 30 minutes daily. The children's awareness level was assessed before and after the snake and ladder game intervention. The collected data were analyzed by various statistical methods.

## Results:

The study samples include 60 school aged children. Out of 60, 28(46.67%) were between 6 to 8 years of age, 19 (31.67%) were 8-10 years of age and 13 (21.67%) were 10-12 years of age. 35(58.33%) were boys and 25 (41.67%) were girls. 32(53.33%) students belongs to Hindu religion, 15(25%) belongs to Muslim and 13 (21.67%) were from other religions.17(28.33%) students' mother has primary education, 17(28.33%)students’ mother has Higher secondary education and 26(43.33%) students' mothers are graduates.8(13.33%) students' father has primary education, 21(35%)students' father has Higher secondary education and 31(53.67%) students' fathers are graduates. In the section of family income, 7 (11.67%) students were under 5000/- per month, 13 (21.67%) were 5000-10,000/- per month, 16 (26.67%)were 10,000-20,000 per month and 24(40%) were above 20,000/- per month. 50% were residing in rural area and 50% were in urban area. 29(48.33%) students had one sibling, 18(30%) students had two siblings and 13(21.67%) students had 3 or more sibling. Figure 1(see PDF) shows in pre test, out of 60 samples, 35 children had average awareness, 15 had good awareness and 10 had very good level of awareness on health habits. After completing the 10 days snake & ladder game intervention, the score level has upgraded to 32 had good, 16 very good and 12 had average awareness regarding health habits. Table 1(see PDF) show that the average awareness scores before the test was 13.83, and after it were 18.63. At the 0.05 level, the computed t value of 21.24 at 59 degrees of freedom was significant. It suggests that when a game-based teaching programme using the snake and ladder game was implemented, students' knowledge of healthy practices in schools was raised. Table 2(see PDF) shows the association of pretest awareness scores of school children on healthy habits with demographic variables age, sex, religion, monthly income, number of siblings, area of residence were not having significant association. While the education status of parents shown significant association with the children's awareness.

## Discussion:

The present study aimed to evaluate the effectiveness of Modified Snake & Ladder Game in improving awareness on health habits among school children. The result analysis showed that Snake & Ladder Game was significantly effective in boosting the awareness level of school children. The findings of this study are supported by another research project conducted in Coimbatore in 2016. According to their study's findings, the mean score for personal hygiene before and after education was 13.68 and 33.31, respectively, and the standard deviation was 5.61 and 6.62, with a mean difference of 19.63. At the 0.001 level of significance, the estimated t value (19.62) was bigger than the table value. Thus, it was determined that the snake and ladder game was successful in improving students' awareness of personal hygiene [9]. Another study done in Nigeria examined the possibility of a board game called Worms and Ladders to complement treatment and lower re-infection rates. According to their research, compared to other kids who played another game, the worm load considerably decreased in kids who played the newly produced game. Children's practices, attitudes, and knowledge all greatly improved. Therefore, the board game Worms and Ladders has the potential to encourage excellent hygiene habits, which would then translate to a lower incidence of illnesses. These results demonstrate the newly created game as a trustworthy supplement to schoolchildren's hygiene routines [10]. In 2018 saw the completion of a study to determine the impact of the snake and ladder game on schoolchildren's awareness of a balanced diet at Pune, Maharashtra. It has been noted that after instruction, the average knowledge score increases to 17.38 from 9.68 before therapy. At a 5 percent level of significance and 99 percent freedom, the researcher can draw the conclusion that playing the game significantly raises the average knowledge score. As a result, the researcher concluded that the Snake and Ladder game is effective at enhancing students' knowledge and skills. [11]. These worldwide research evidences strongly recommends that Snake & Ladder Game can be utilized as an effective method of health education for school aged children. The results of the current study showed a substantial relationship between children's health behaviours and their parents' educational attainment. At the 0.05 level of significance, it was determined that the Chi square values for the mother's and fathers educational statuses were significant. Another study by Kamala Devi that demonstrated a relationship between the father's educational status and the children's prior knowledge of personal cleanliness (Chi square value: 44.05) supports this finding [9]. Another study that looked at how parental education levels affected students' eating patterns in Nigerian elementary schools found that children with low parental education levels have less healthy eating habits than kids with high parental education levels. Their findings add to the body of literature demonstrating how parents' educational levels differentially influence children's healthy eating patterns. Through nutritional education and counselling, which have been proposed as effective approaches in this context, interventions intended to change these eating behaviours should concentrate on the parental involvement in the development of eating patterns [12].

## Conclusion:

The present study identified that the children's awareness level on healthy habits was increased after undergoing snake & ladder game intervention. The awareness of healthy behaviors among schoolchildren scored lower on the pretest. After administering the snake and ladder game regarding healthy practices, it was discovered that the post test score had significantly increased. Therefore the investigators concluded that Modified Snake & Ladder Game can be utilized effectively for educating school aged children regarding health related topics.
